# PD-1^+^ and TIM-3^+^ T cells widely express common γ-chain cytokine receptors in multiple myeloma patients, and IL-2, IL-7, IL-15 stimulation up-regulates PD-1 and TIM-3 on T cells

**DOI:** 10.32604/or.2024.047893

**Published:** 2024-09-18

**Authors:** EGOR V. BATOROV, ALISA D. INESHINA, TATIANA A. ARISTOVA, VERA V. DENISOVA, SVETLANA A. SIZIKOVA, DARIA S. BATOROVA, GALINA Y. USHAKOVA, EKATERINA Y. SHEVELA, ELENA R. CHERNYKH

**Affiliations:** 1 Laboratory of Cellular Immunotherapy, Research Institute of Fundamental and Clinical Immunology, Novosibirsk, 630099, Russia; 2 V. Zelman Institute of Medicine and Psychology, Novosibirsk National Research State University, Novosibirsk, 630090, Russia; 3 Department of Hematology and Bone Marrow Transplantation, Research Institute of Fundamental and Clinical Immunology, Novosibirsk, 630099, Russia

**Keywords:** Autologous hematopoietic stem cell transplantation (AHSCT), CD25, CD122, Eomesodermin (EOMES), Homeostatic proliferation

## Abstract

**Background:**

Immune checkpoint ligand-receptor interactions appear to be associated with multiple myeloma (MM) progression. Simultaneously, previous studies showed the possibility of PD-1 and TIM-3 expression on T cells upon stimulation with common γ-chain family cytokines *in vitro* and during homeostatic proliferation. The aim of the present work was to study the impact of homeostatic proliferation on the expansion of certain T cell subsets up-regulating PD-1 and TIM-3 checkpoint molecules.

**Methods:**

The expression of CD25, CD122, CD127 common γ-chain cytokine receptors, phosphorylated signal transducer and activator of transcription-5 (pSTAT5) and eomesodermin (EOMES) was comparatively assessed with flow cytometry in PD-1- and TIM-3-negative and positive T cells before the conditioning and during the first post-transplant month in peripheral blood samples of MM patients.

**Results:**

Substantial proportions of PD-1- and TIM-3-positive T lymphocytes expressed common γ-chain cytokine receptors and pSTAT5. Frequencies of cytokine receptor expressing cells were significantly higher within TIM-3^+^ T cells compared to PD-1^+^TIM-3^−^ subsets. Considerable proportions of both PD-1-/TIM-3-negative and positive CD8^+^ T cells express EOMES, while only moderate frequencies of CD4^+^ PD-1^+^/TIM-3^+^ T cells up-regulate this transcription factor. Besides, the surface presence of CD25 and intranuclear expression of EOMES in CD4^+^ T cells were mutually exclusive regardless of PD-1 and TIM-3 expression. The stimulation with common γ-chain cytokines up-regulates PD-1 and TIM-3 during the proliferation of initially PD-1/TIM-3-negative T cells but fails to expand initially PD-1^+^ and TIM-3^+^ T cell subsets *in vitro*.

**Conclusions:**

Both PD-1 and TIM-3 expressing T cells appear to be able to respond to homeostatic cytokine stimulation. Differences in common γ-chain cytokine receptor expression between PD-1^+^ and TIM-3^+^ T cells may reflect functional dissimilarity of these cell subsets. Checkpoint blockade appears to alleviate lymphopenia-induced proliferation of PD-1^+^ T cells but may raise the possibility of immune-mediated adverse events.

## Introduction

Multiple myeloma (MM) is a hematologic neoplasm of differentiated monoclonal plasma cells [1]. Both the prevalence and survival rates of MM have increased over the past 20 years [2]. Despite the utilization of proteasome inhibitors, immunomodulatory drugs (IMiDs), different types of biological therapy and high-dose melphalan conditioning with subsequent autologous hematopoietic stem cell transplantation (AHSCT), MM is considered an incurable disease.

Only two monoclonal antibodies (MAbs), anti-CD38 and anti-SLAMF7, have demonstrated efficacy for the treatment of MM. While anti-PD-1/PD-L1 MAbs had been used successfully to treat various solid malignancies and Hodgkin lymphoma, monotherapy with nivolumab or pembrolizumab for relapsed and refractory MM (RRMM) failed to achieve an objective response [3,4]. Besides, pembrolizumab in combination with IMiDs/dexamethasone in patients with untreated MM and RRMM showed a high frequency of immune-mediated adverse events and a decrease in survival rates [5,6]. Other immune checkpoint inhibitors—against TIM-3, LAG-3, etc., may be promising, but in the case of MM, these MAbs are in the early stages of research.

Poor responses to anti-PD-1 therapy in MM may be due not to the restoration of exhausted T cell functions, but to the dysregulation of activated PD-1^+^ T cells, e.g., virus-specific or autoreactive populations. Functional characteristics of T lymphocytes up-regulating different checkpoint molecules also differ. Apparently, TIM-3^+^ T cells are more exhausted than the PD-1^+^ compartment, which seems to retain cytotoxic and cytokine-producing potential [7]. In addition, different studies showed the possibility of PD-1 and TIM-3 up-regulation on T lymphocytes upon stimulation with cytokines belonging to the common cytokine receptor γ-chain family (interleukin-2 (IL-2), IL-7, IL-15, IL-21) *in vitro* and during homeostatic proliferation *in vivo* [8–10]. The letter is of interest, as the frequencies of PD-1- and TIM-3-positive T cells were incremented at early post-transplant [10–12] and several researchers supposed that it would be an appropriate period for targeted anti-checkpoint therapy [10,13,14]. Besides the increased proliferative capacity, we and other authors previously had shown sustained cytotoxic potential of PD-1-expressing T cells during the post-transplant immune recovery period [11,12].

Nonetheless, it remains uncertain whether homeostatic proliferation induces the appearance of surface PD-1 and TIM-3 on initially non-expressing T lymphocytes without a loss of effector functions or pre-existing reinfused PD-1^+^ and TIM-3^+^ subsets restore proliferative and perforin-producing ability under cytokine-mediated immune activation.

To assess the impact of homeostatic proliferation on the expansion of certain T cell subsets expressing distinct inhibitory checkpoint receptors, we comparatively studied common γ-chain receptors and associated intracellular molecules up-regulated in PD-1- and PD-1- TIM-3- expressing T cells before the conditioning and closely after AHSCT in MM patients. In addition, we evaluated an ability to express surface PD-1 and TIM-3 receptors by initially negative and positive T lymphocytes under stimulation through T cell receptor (TCR) or with homeostatic cytokines *in vitro*.

## Patients, Materials, and Methods

### Patients and healthy donors

The prospective study included 53 MM patients who had received the conditioning with high-dose melphalan (140–200 mg/m^2^) and AHSCT and 16 matched healthy controls. All participants provided informed consent in line with the 1975 Helsinki Declaration. The study protocol was approved by the Research Institute of Fundamental and Clinical Immunology Ethics Committee (No. 9; 25 August, 2021). Patient characteristics are listed in Table 1. Prior to the conditioning, the patients had been treated with standard chemotherapy regimens. For hematopoietic stem cell (HSC) mobilization, the patients received either high-dose cyclophosphamide (4 g/m^2^) or, occasionally, a regular course of chemotherapy followed by injections of granulocyte colony-stimulating factor (5 μg/kg/day). The median dose of administered CD45^+^CD34^+^ HSCs was 4.80 × 10^6^/kg (3.10–5.70 × 10^6^/kg).

**Table 1:** Patient baseline characteristics

Characteristic	Number (%)
Age at AHSCT, years; median (min-max)	50, 5 (35–65)
Sex	
Female	27 (51%)
Male	26 (49%)
Types of paraproteins	
IgG kappa	24 (45%)
IgG lambda	8 (15%)
IgA kappa	5 (9%)
IgA lambda	3 (6%)
Light chain	7 (14%)
Not available	6 (11%)
Durie-Salmon stage	
II	19 (36%)
III	34 (64%)
Status before HD Mel with AHSCT	
- 1st CR	19 (36%)
- PR, VGPR, 2nd CR	33 (62%)
- SD	1 (2%)
Months between diagnosis and HD Mel with AHSCT; median (interquartile range)	11.5 (8.7–14.3)
Pre-transplant therapy	
1 (bortezomib-based regimens)	37 (70%)
2 (bortezomib-based regimens + DCEP/EDAP or lenalidomide-containing regimens)	12 (23%)
≥3 (bortezomib-based regimens + DCEP/EDAP + lenalidomide-containing regimens)	4 (7%)

Note: The International Myeloma Working Group’s criteria were followed in defining the responses. Abbreviations: AHSCT, autologous hematopoietic stem cell transplantation; CR, complete remission; DCEP, dexamethasone, cyclophosphamide, etoposide, platinol; EDAP, etoposide, dexamethasone, ara-C, platinol; HD Mel, high-dose melphapan; PR, partial response; SD, stable disease; VGPR, very good partial response.

### Blood samples

Peripheral blood (PB) specimens were collected from the patients as part of standard diagnostic procedures before melphalan administration (on day −4; *n* = 49) and at the engraftment (on average, day +14; *n* = 53). After being collected, PB specimens were processed in two hours. Density gradient centrifugation was performed to obtain mononuclear cells (MNCs). Briefly, 5–8 mL of whole blood were carefully layered onto 3 mL of Ficoll solution (ρ = 1.077 g/mL), centrifuged at 3000 RPM for 20 min, MNCs were aspirated from the interface. After two rounds of washing in phosphate buffer solution, MNCs were subjected to flow cytometry analysis.

### Flow cytometry analysis

**Figure 1:**
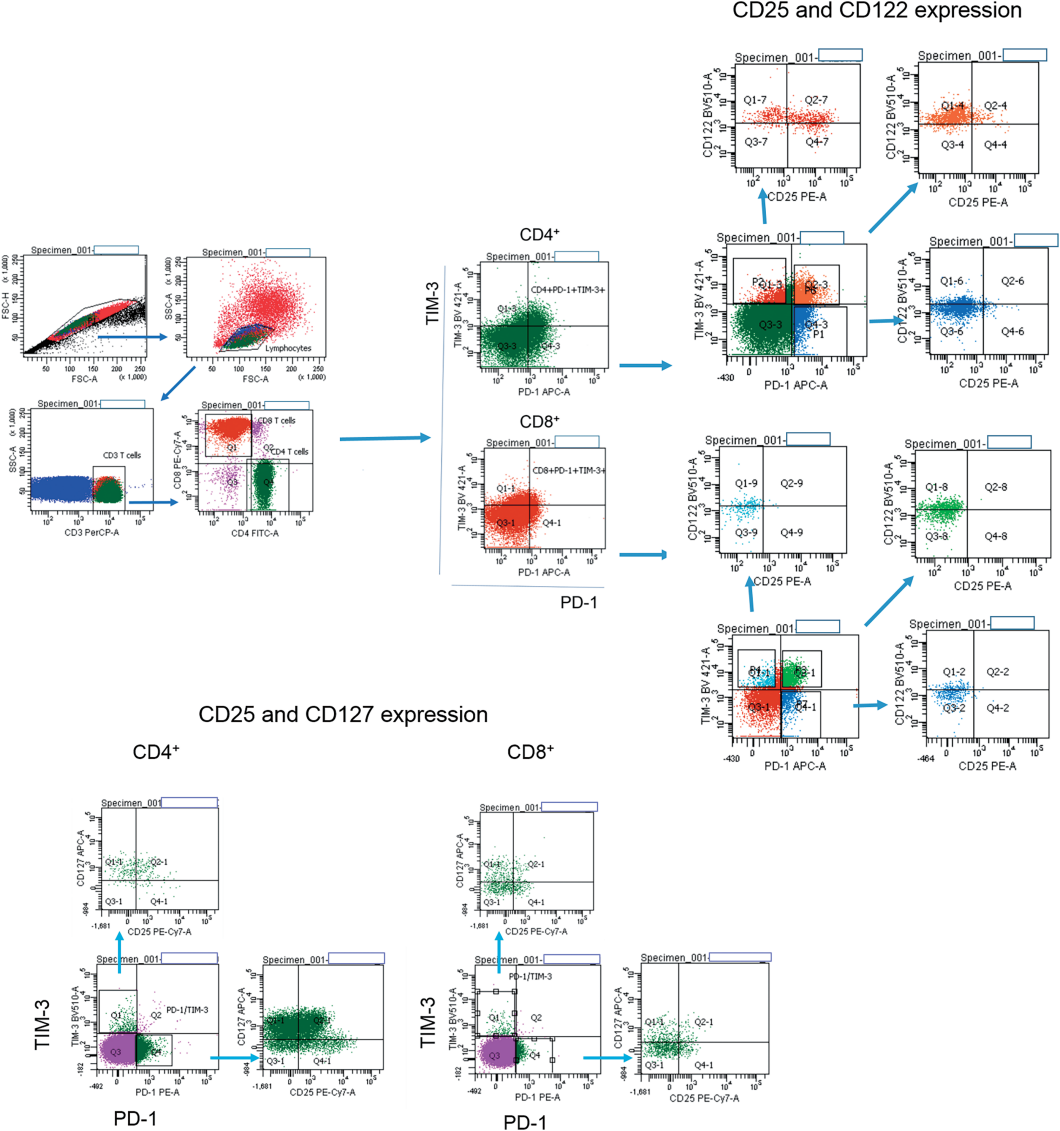
Flow cytometry gating strategies. Gating strategies for PD-1^+^ and TIM-3^+^ T lymphocytes expressing CD25, CD122, CD127 are presented.

Surface and intracellular staining of MNCs was performed using the following anti-human monoclonal antibodies: anti-CD3 (FITC; BD Biosciences, San Jose, CA, USA; PerCP; Biolegend, San Diego, CA, USA), anti-CD4 (PerCP; BD), anti-CD8 (FITC, PE-Cy 7; BD), anti-CD25 (FITC, PE-Cy 7; BD), anti-CD122 (BV510; BD), anti-CD127 (Alexa Fluor 647; BD), anti-PD-1 (PE, APC; BD) and anti-TIM-3 (PE, BV421; BD; PerCP-Cy 5.5; Biolegend), anti-Ki-67 (PE), anti-pSTAT5 (PE), anti-eomesodermin (EOMES) (PE; eBioscience, San Diego, CA, USA). The expression of CD25, CD122, CD127 and nuclear proteins Ki-67, pSTAT5, EOMES were assessed in PD-1^−^TIM-3^−^, PD-1^+^TIM-3^−^, TIM-3^+^PD-1^−^, PD-1^+^TIM-3^+^ T cell subsets. Fig. 1 shows the flow cytometric gating approach. Samples were first processed with antibodies against surface molecules, and then incubated with fixation and permeabilization solutions (BD Pharmingen™ Transcription Factor Buffer Set, BD Biosciences) and finally stained with anti-Ki-67 or anti-EOMES monoclonal antibodies. To assess intracellular expression of phosphorylated signal transducer and activator of transcription-5 (pSTAT5), MNCs were incubated with recombinant IL-2 (BIOTECH, St. Petersburg, Russia) (100 ng/mL of cell suspension, 10^6^ cells/mL) for 15 min. After the incubation, cell suspension was fixed with BD Phosflow™ Fix Buffer I and permeabilized with BD Phosflow™ Perm Buffer III (contains 87.68% methanol) (BD Biosciences) for 30 min on ice.

BD™ CompBeads Anti-mouse Ig, κ/Negative Control Compensation Particles Set (BD Biosciences), unstained live cells, “fluorescence-minus-one” were used as controls. Staining with 7-Aminoactinomycin D was performed to assess the cell viability. Prepared samples were examined on the FACSCanto II flow cytometer with FACSDiva software (BD Biosciences). At least 50,000 events in the CD3^+^CD8^+^ gate were collected for the assay.

### Isolation of T cell subsets

**Figure A1:**
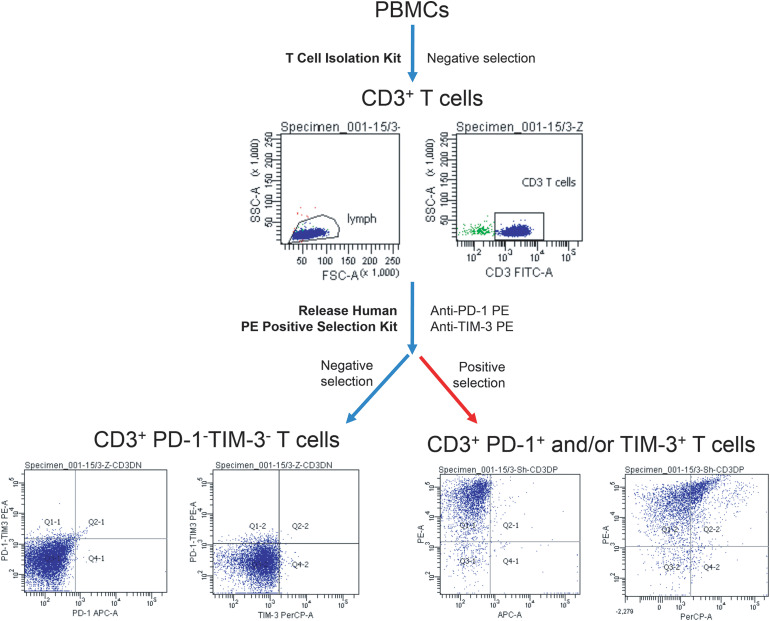
Sequential bead magnetic selection of PD-1- and TIM-3-negative and PD-1/TIM-3-positive CD3^+^ T cells for *in vitro* culture. Strategy of immunomagnetic bead selection of PD-1^−^TIM-3^−^ and CD3^+^ PD-1^+^ and/or TIM-3^+^ T lymphocyte subsets of representative MM patient is presented.

CD3^+^ T cells were isolated from PB MNCs of eight MM patients using negative immunomagnetic bead selection with EasySep™ Human T Cell Isolation Kit (STEMCELL Technologies, Vancouver, BC, Canada). Then, obtained CD3^+^ T cells were labeled with anti-PD-1 and anti-TIM-3 PE-conjugated monoclonal antibodies and divided into PD-1^−^TIM-3^−^ (negative selection) and PD-1^+^ or TIM-3^+^ (positive selection) subsets using EasySep™ Release Human PE Positive Selection Kit (STEMCELL Technologies, Vancouver, BC, Canada). The isolation strategy is presented in Fig. A1.

### In vitro cytokine-induced expansion of PD-1^+^ and TIM-3^+^ T cells

The isolated PD-1^−^TIM-3^−^ or PD-1^+^/TIM-3^+^ T cell subsets (0.4–1.0 × 10^6^ cells) were cultured separately in 96-well round-bottom plates (10^5^/well) at RPMI-1640 medium (Sigma-Aldrich, St. Louis, MO, USA) containing 10% autologous blood plasma alone (negative control) or with anti-CD3 monoclonal antibodies (3 μg/mL) (MedBioSpectr, Moscow, Russia) + IL-2 (50 U/mL) (BIOTECH, St. Petersburg, Russia), as a positive control, or with combination of cytokines: IL-2 (50 U/mL) + IL-7 (50 ng/mL) + IL-15 (50 ng/mL) (both purchased from SCI-Store, Moscow, Russia). Proportions of PD-1- and TIM-3-expressing CD4^+^ and CD8^+^ T cells were assessed using anti-PD-1 (APC) and TIM-3 (PerCP-Cy 5.5) monoclonal antibodies following 7 days of cultivation.

### Statistical analysis

The Statistica 6 program (StatSoft, Inc., Tulsa, OK, USA) was used to conduct the statistical analysis. Unless otherwise noted, the data were shown as the median and interquartile ranges. The Mann-Whitney U test was applied to compare differences between two independent groups. To calculate differences in parameters among the paired groups, the sign test was utilized. The *p* values presented in the text were for two-tailed tests. *p* less than 0.05 was regarded as statistically significant. GraphPad Prism 5 software (GraphPad Software, Inc., La Jolla, CA, USA) was used to create the graphs.

## Results

### Early increase in PB PD-1^+^ and TIM-3^+^ T cells of MM patients following AHSCT

**Figure 2:**
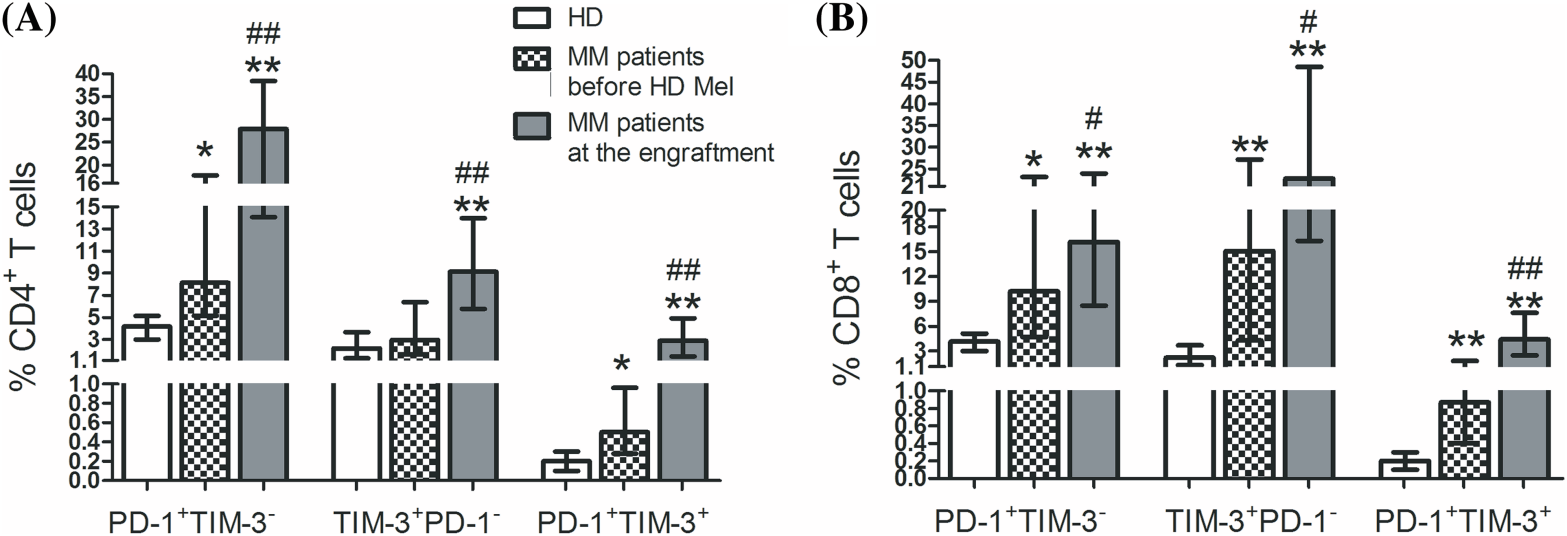
Frequencies of circulating PD-1^+^ and TIM-3^+^ T lymphocytes of patients with multiple myeloma and healthy controls. PD-1^+^TIM-3^−^, TIM-3^+^PD-1^−^, PD-1^+^TIM-3^+^ subsets of CD4^+^ (А) and CD8^+^ (В) T lymphocytes in PB samples obtained from healthy donors (HD, empty bars; *n* = 16), MM patients before the conditioning with high-dose melphalan (before HD Mel, hatched bars; *n* = 49) and during the first month following AHSCT (engraftment, plain gray bars; *n* = 53) are showed. Statistical differences are evaluated with the Mann–Whitney U-test. Asterisks are used to indicate significant differences between healthy donors and MM patients (*p < 0.001, **p < 0.0001). Significant differences between MM patients before the conditioning with high-dose melphalan and during the first month following AHSCT are specified as number signs (^#^*p* < 0.05, ^##^*p* < 0.0001).

We first assessed frequencies of circulating T cell subsets up-regulating separately PD-1 or TIM-3 and co-expressing both receptors in healthy individuals and MM patients prior to the conditioning and during the 1st month after AHSCT. PD-1^+^ and TIM-3^+^ subsets of CD4^+^ and CD8^+^ T cells were significantly higher in patients than in healthy donors (except TIM-3^+^ subset of CD4^+^ T cells prior to the conditioning, *p* = 0.17). Frequencies of studied PD-1- and/or TIM-3-expressing T cells increased dramatically in the 1st month after AHSCT compared to the pre-transplant values (Fig. 2), which agreed with previous findings [10–12].

**Figure 3:**
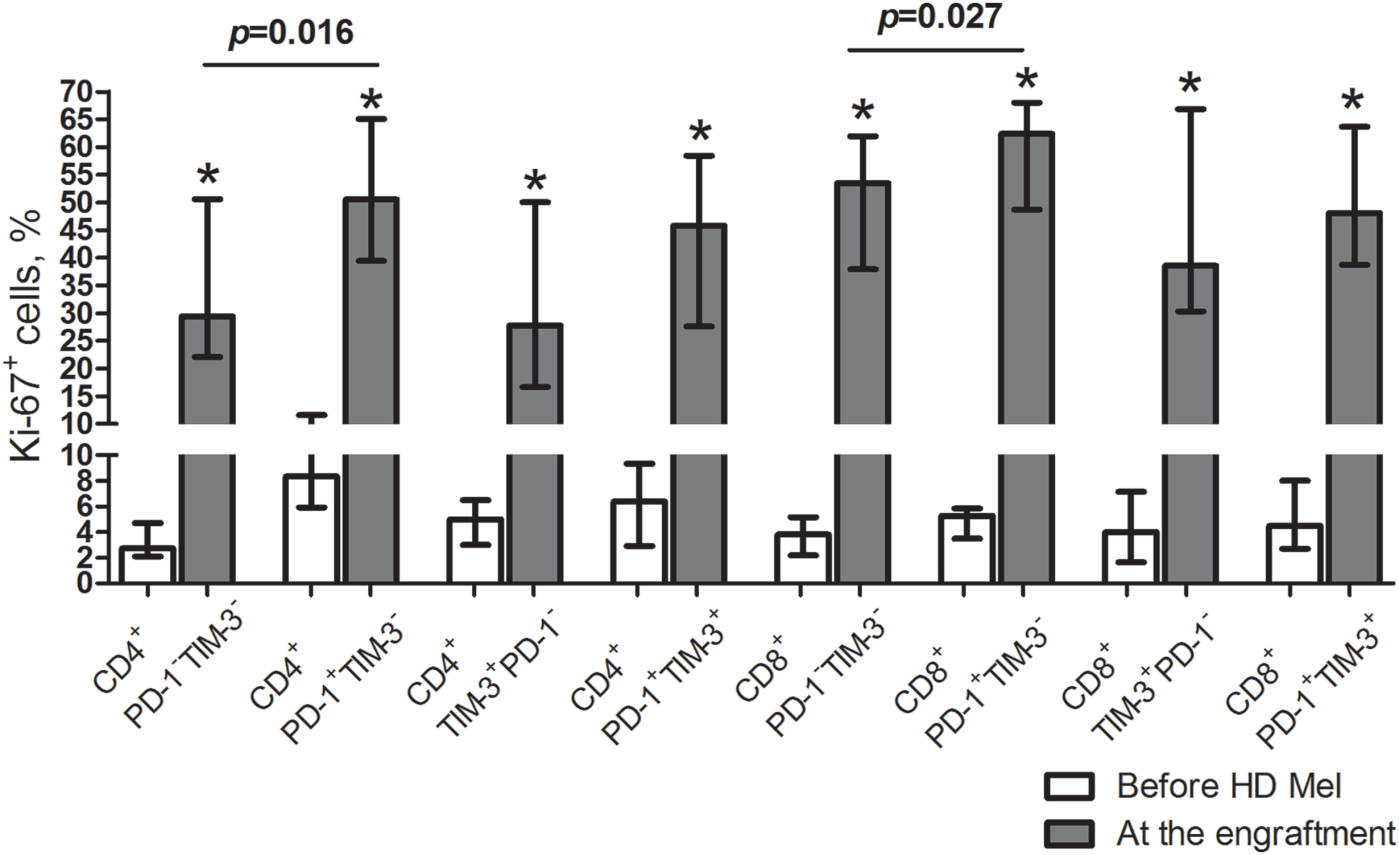
Frequencies of proliferating cells in circulating PD-1- and TIM-3-expressing and non-expressing T lymphocytes of MM patients prior to the conditioning and at the early post-transplant. Proportions of Ki-67^+^ cells in PD-1^−^TIM-3^−^, PD-1^+^TIM-3^−^, TIM-3^+^PD-1^−^ and PD-1^+^TIM-3^+^ CD4^+^ and CD8^+^ T cell subsets in PB samples of patients prior to the conditioning with high-dose melphalan (before HD Mel, empty bars; *n* = 15) and during the first month following AHSCT (plain grey bars; *n* = 12) are presented. *p* values between MM patients prior to the conditioning with high-dose melphalan and during the first month following AHSCT are evaluated with the Mann–Whitney U-test (*p < 0.005). Significant differences between certain cell subsets are evaluated with the sign test (*p*) and presented in the figure.

At post-transplant, T cell subsets up-regulated Ki-67, a marker of cell proliferation, regardless of PD-1 and/or TIM-3 expression. Relative counts of Ki-67-expressing cells were significantly higher in PD-1^+^TIM-3^−^ T cells (but not in TIM-3^+^ subsets) compared to PD-1/TIM-3-negative “conventional” T cells (Fig. 3). So, there is an increment in the proportions of actively proliferating T lymphocytes up-regulating PD-1 and TIM-3 at the early post-transplant, presumably mediated by homeostatic proliferation.

### Expression of common γ-chain cytokine receptors in PD-1^+^ and TIM-3^+^ T lymphocytes of MM patients

**Figure 4:**
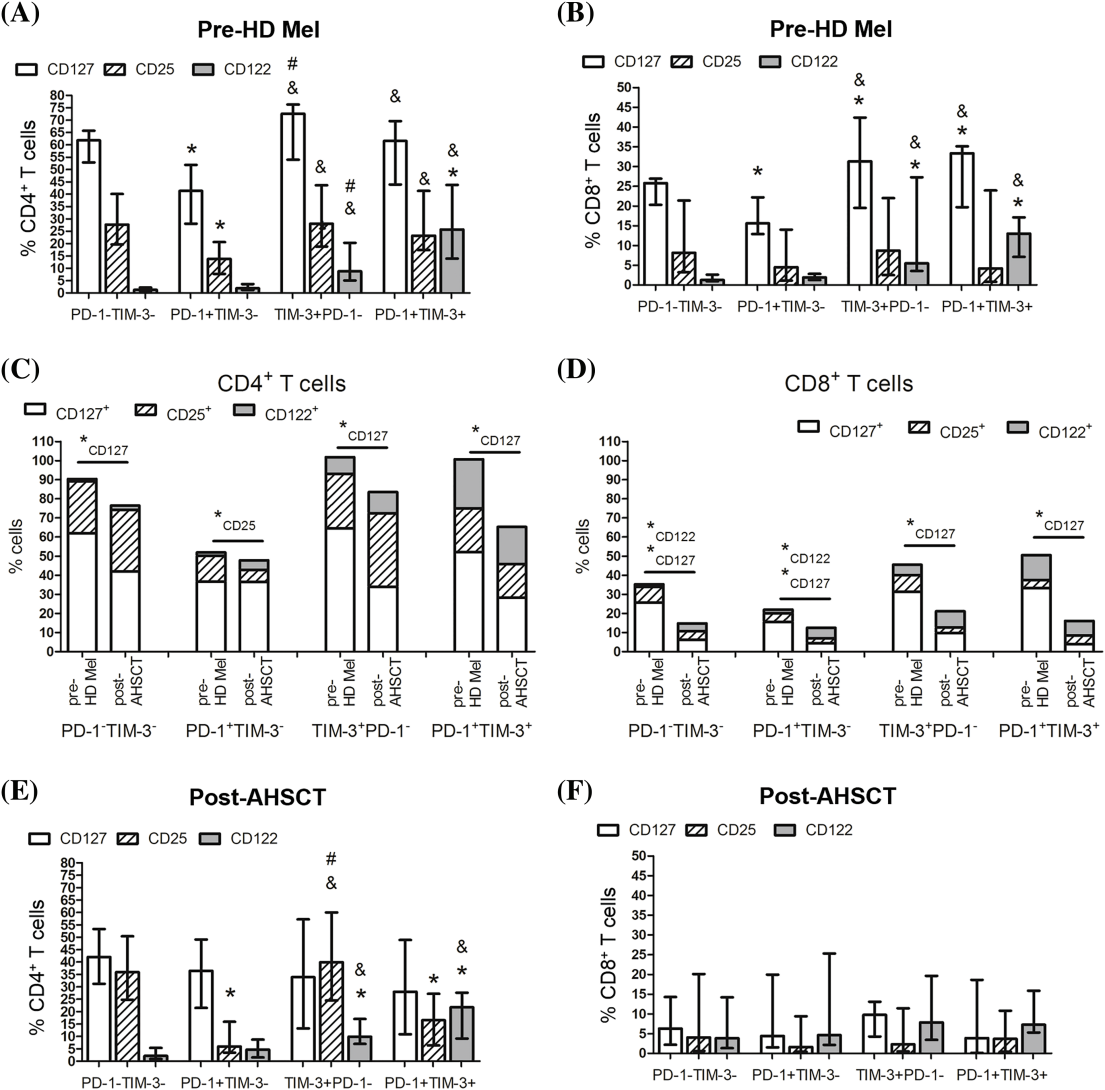
Surface expression of homeostatic cytokine receptors in circulating PD-1- and TIM-3-negative and positive T lymphocytes of MM patients. Proportions of CD25^+^, CD122^+^, CD127^+^ cells in PD-1^−^TIM-3^−^, PD-1^+^TIM-3^−^, TIM-3^+^PD-1^−^, PD-1^+^TIM-3^+^ CD4^+^ (A, C, D) and CD8^+^ (B, E, F) T lymphocytes in PB samples of patients prior to the conditioning with high-dose melphalan (A–C, E, pre-HD Mel; *n* = 22) and during the first month following AHSCT (A, B, D, F, post-AHSCT; *n* = 21) are presented. (A, B) Data in stacked bars are presented as medians. Statistical differences are evaluated with the Mann–Whitney U-test (*p_*U*_ < 0.05), and statistically changed subsets are flagged as subscripts under the asterisks. (C–F) Data are expressed as median and interquartile range. Statistical differences are evaluated with the sign test. Statistical differences are showed comparing with PD-1^−^TIM-3^−^ cells (*p < 0.05), PD-1^+^TIM-3^+^ cells (^#^*p* < 0.05) and PD-1^+^TIM-3^−^ cells (^&^*p* < 0.05).

Cytokines driving homeostatic expansion exert their effects by binding to specific receptors on lymphocytes. We studied the expression of common γ-chain receptors CD25, CD122, CD127 to assess the functional state and responsiveness of PD-1^+^ and TIM^+^ T lymphocytes under the conditions of lymphopenia-induced proliferation. Before the conditioning, substantial proportions of the studied subsets were CD127-positive. Frequencies of CD25^+^ (CD4^+^ T cells only), CD122^+^, CD127^+^ cells were significantly higher within TIM-3-positive (including co-expressing PD-1) CD4^+^ and CD8^+^ T lymphocytes compared to PD-1^+^TIM-3^−^ subsets (Fig. 4A,B).

Almost all PD-1- and TIM-3-negative and positive T cells downregulated the expression of CD127 at early post-transplant compared with their pre-conditioning levels, in particular, within the CD8^+^ cell compartment. Simultaneously, CD122^+^ cells were significantly increased in PD-1^−^TIM-3^−^ and PD-1^+^TIM-3^−^ CD8^+^ T cells. The only change in PD-1^+^TIM-3^−^ a subset of CD4^+^ T cells was the decreased frequency of CD25^+^ cells at the engraftment (Fig. 4C,D).

During the first month following AHSCT, CD4^+^ PD-1^−^TIM-3^−^ “conventional” T cells and CD4^+^ TIM-3^+^PD-1^−^ subset contained the highest proportions of CD25^+^ cells (32.2 (23.5%–49.4%) and 38.6 (24.1%–57.9%), *n* = 21, respectively), while CD25 expression was relatively rare in CD4^+^ PD-1^+^TIM-3^−^ subset (6.3% (3.5%–15.0%), *n* = 21) (Fig. 4D). The frequencies of CD122^+^ cells retained significantly higher within TIM-3-positive subsets (TIM-3^+^PD-1^−^ and PD-1^+^TIM-3^+^) of CD4^+^ T cells compared with PD-1^−^TIM-3^−^ and PD-1^+^TIM-3^−^ subsets as before the conditioning (Fig. 4E). The relatively low proportion of CD8^+^ T lymphocytes expressed the evaluated cytokine receptors (Fig. 4F).

Thus, in MM patients, substantial proportions of CD4^+^ T lymphocytes express common γ-chain cytokine receptors irrespectively of PD-1 and TIM-3 expression, especially before the conditioning. CD8^+^ T lymphocytes express moderate to low levels of homeostatic cytokine receptors. At the engraftment, a sufficient decrease in frequencies of CD127^+^ cells coincides between PD-1^−^TIM-3^−^ “conventional” T cells and PD-1- and/or TIM-3-positive subsets (except PD-1^+^TIM-3^−^ CD4^+^ T cells). In general, TIM-3-positive subsets of CD4^+^ and CD8^+^ (at pre-transplant only) T cells express higher levels of cytokine receptors compared to the PD-1^+^TIM-3^−^ compartment.

### Dynamics of intracellular expression of the transcription factors STAT5 and EOMES in circulating PD-1- and TIM-3-expressing T lymphocytes of MM patients following AHSCT

**Figure 5:**
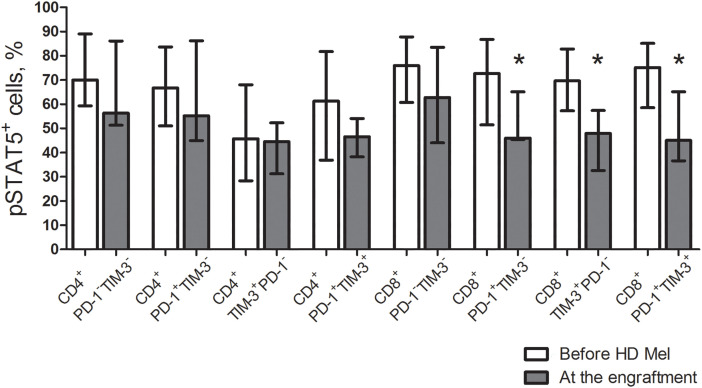
Frequencies of pSTAT5^+^ cells in circulating PD-1- and TIM-3-expressing and non-expressing T lymphocytess of MM patients prior to the conditioning and following AHSCT. Proportions of cells expressing phosphorylated STAT5 (pSTAT5) in PD-1^−^TIM-3^−^, PD-1^+^TIM-3^−^, TIM-3^+^PD-1^−^ and PD-1^+^TIM-3^+^ CD4^+^ and CD8^+^ T cell subsets in PB samples of patients prior to the administration of high-dose melphalan (before HD Mel, empty bars; *n* = 10) and at the engraftment day (plain grey bars; *n* = 8) are presented. *p* values between MM patients prior to the administration of high-dose melphalan and during the 1st month after AHSCT are evaluated with the Mann–Whitney U-test (*p < 0.05).

The studied cytokine receptors transduce signals via the Janus kinase—signal transducer and activator of the transcription-5 (STAT5) pathway. In turn, STAT5 activation is crucial for cytokine-induced T cell expansion and expression of common γ-chain cytokine receptors. Therefore, we further investigated intracellular phosphorylated STAT5 (pSTAT5) in the studied T cell subsets. Prior to the conditioning, the frequencies of pSTAT5^+^ T cell subsets were not different regardless of PD-1 and TIM-3 expression. After AHSCT, proportions of pSTAT5^+^ cells in all studied CD4^+^ T cell subsets and PD-1^−^TIM-3^−^ “conventional” CD8^+^ T cells remained unaltered from their pre-conditioning counts, while PD-1- and TIM-expressing CD8^+^ T cells significantly downregulated pSTAT5 at early post-transplant (Fig. 5).

**Figure 6:**
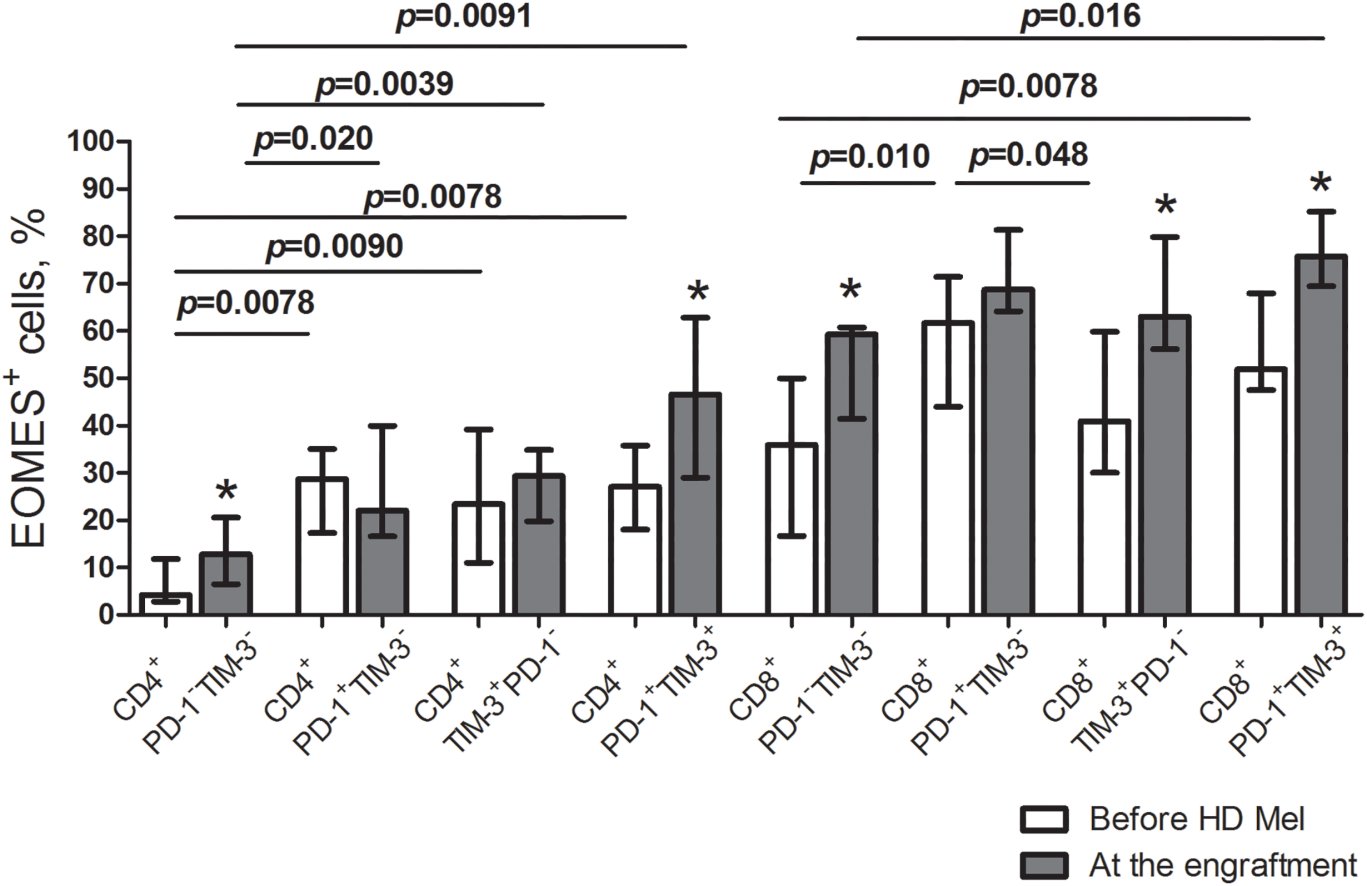
EOMES^+^ subsets in circulating PD-1- and TIM-3-expressing and non-expressing T lymphocytes of MM patients prior to the conditioning and following AHSCT. Relative counts of EOMES^+^ cells in PD-1^−^TIM-3^−^, PD-1^+^TIM-3^−^, TIM-3^+^PD-1^−^ and PD-1^+^TIM-3^+^ CD4^+^ and CD8^+^ T cell subsets in PB samples of patients prior to the administration of high-dose melphalan (before HD Mel, empty bars; *n* = 10) and at the engraftment day (plain grey bars; *n* = 8) are presented. *p* values between MM patients prior to the administration of high-dose melphalan and during the 1^st^ month after AHSCT are evaluated with the Mann–Whitney U-test (*p < 0.05). Significant differences between certain cell subsets are evaluated with the sign test (*p*) and presented in the figure.

The transcription factor eomesodermin (EOMES) is known implicated in both T cell exhaustion and activation. We evaluated EOMES expression in the listed T cell subsets. Before AHSCT, EOMES-positive CD4^+^ PD-1^−^TIM-3^−^ T cells were low (4.6% (2.7%–11.1%); *n* = 10), while the frequencies of EOMES^+^ cells were significantly higher between PD-1^+^/TIM-3^+^ CD4^+^ T cell subsets. Contrary to their CD4^+^ counterparts, a substantial part of PD-1^−^TIM-3^−^ “conventional” CD8^+^ T cells expressed EOMES (39.4% (17.9%–51.7%); *n* = 10). EOMES^+^ cells were the most abundantly present in PD-1^+^TIM-3^−^ CD8^+^ T lymphocytes (65.4% (43.5%–88.4%); *n* = 10), which was significantly higher compared with PD-1/TIM-3-negative and TIM-3^+^PD-1^−^ CD8^+^ T cells (Fig. 5). At the engraftment, relative counts of EOMES-expressing cells were significantly increased in PD-1/TIM-3-negative and PD-1^+^TIM-3^+^ CD4^+^ and CD8^+^ T lymphocytes and TIM-3^+^PD-1^−^ CD8^+^ T cell subset compared to pre-transplant values. Due to simultaneous increment in EOMES^+^ cells following AHSCT, PD-1/TIM-3-expressing CD4^+^ T lymphocytes and PD-1^+^TIM-3^+^ CD8^+^ T cell subset remained significantly higher compared with PD-1/TIM-3-non-expressing “conventional” T cells (Fig. 6).

### Mutually exclusive expression of CD25 and EOMES in CD4^+^ T lymphocytes

**Figure 7:**
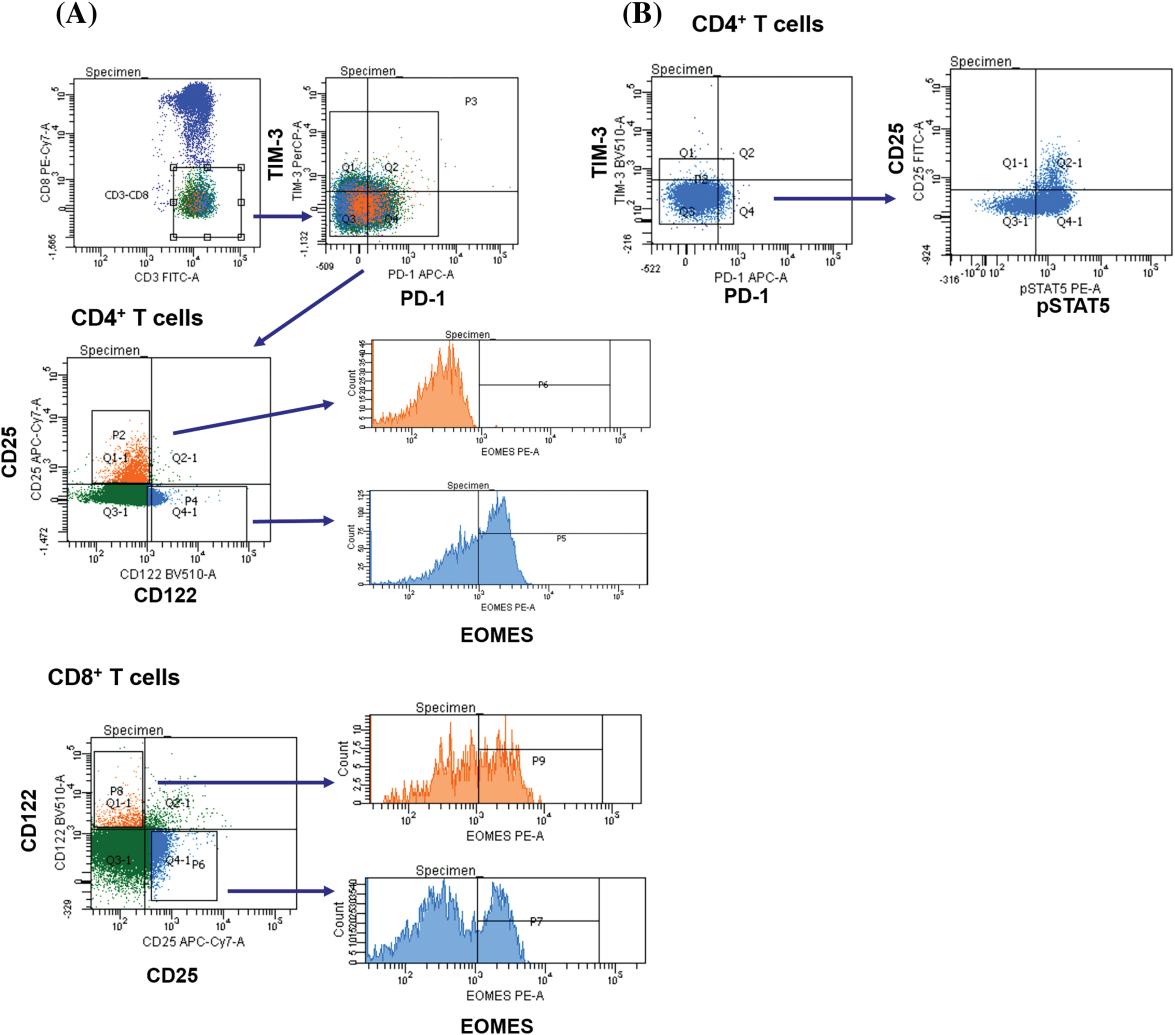
CD4^+^CD25^+^ T cells lack expression of the transcription factor EOMES. Up-regulation of CD25 is associated with the lack of intranuclear expression of EOMES in CD4^+^ T cells. CD8^+^CD25^+^ T cells and 122^+^ T cell subsets express EOMES (A). CD4^+^CD25^+^ T cells co-express pSTAT5 regardless of PD-1 and TIM-3 positivity (B).

When studying the above-mentioned transcription factors, we found that the surface presence of CD25 and intracellular expression of EOMES in CD4^+^ T lymphocytes were mutually exclusive regardless of PD-1 and TIM-3 surface expression (Fig. 7). CD122 and CD127 did not show any such association. Besides, all CD25-expressing T cells were pSTAT5^+^, but not vice versa.

### Homeostatic cytokines stimulate PD-1 and TIM-3 expression on proliferating T cells but fail to expand pre-existing PD-1^+^ and TIM-3^+^ T lymphocytes of MM patients in vitro

**Figure 8:**
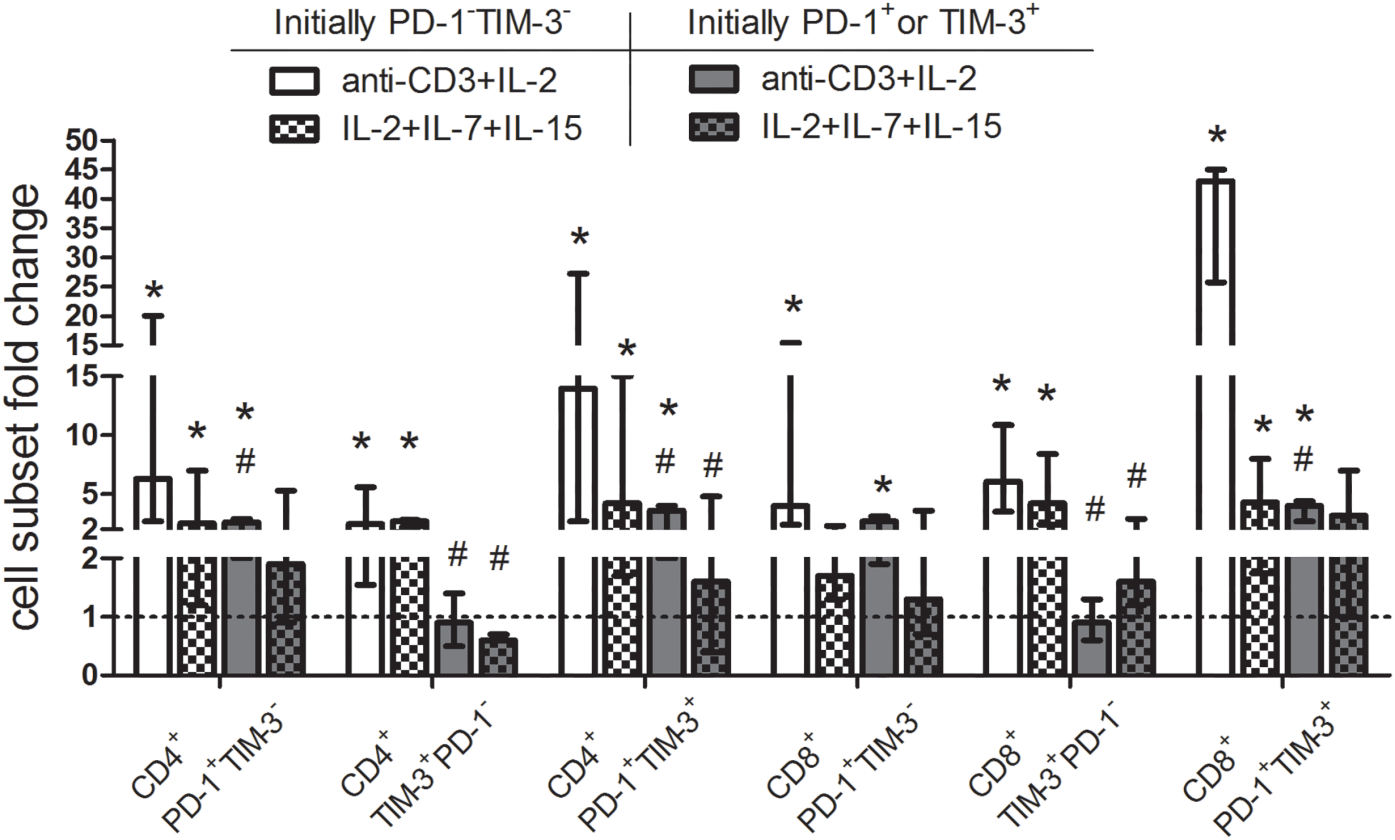
Up-regulation of surface PD-1 and TIM-3 on initially PD-1- and TIM-3-non-expressing T lymphocytes and the lack of expansion of PD-1-/TIM-3-expressing T cells following stimulation with homeostatic cytokines. Purified PD-1^−^TIM-3^−^ and PD-1^+^ or TIM-3^+^ T cells of MM patients (*n* = 8) were cultured with media alone or stimulated separately with anti-CD3 (3 μg/mL) + IL-2 (50 U/mL) or IL-2 (50 U/mL) + IL-7 (50 ng/mL) + IL-15 (50 ng/mL) for 7 days. For each subset, the frequencies in unstimulated T cell cultures were considered as baseline values (dashed line) to calculate fold changes induced by stimulation with anti-CD3+IL-2 or homeostatic cytokines. Note: Data are expressed as median and interquartile range. Statistical significance is evaluated with the Mann–Whitney U-test *p < 0.05 comparing with the frequencies of T cell subsets in unstimulated cultures, ^#^*p* < 0.05 comparing with the frequencies of the same subsets in the initially PD-1/TIM-3-negative cultures.

Further, we studied whether cytokine-mediated stimulation can induce PD-1 and TIM-3 expression on proliferating initially PD-1/TIM-3-negative T cells or cause the expansion of pre-existing PD-1/TIM-3-positive subsets obtained from eight MM patients. PD-1 and TIM-3-non-expressing, PD-1^+^ and TIM-3^+^ CD3^+^ T cells were isolated by a negative and positive magnetic selection, respectively. Isolated cells were cultured with media alone (negative control) or stimulated with the combination of cytokines IL-2, IL-7, IL-15, or, as a positive control, with anti-CD3 and IL-2. In initially PD-1^−^TIM-3^−^ T cells, the stimulation with both anti-CD3+IL-2 and homeostatic cytokines increased the proportions of PD-1^+^ and TIM-3^+^ subsets (except CD8^+^PD-1^+^TIM-3^−^ cells stimulated with interleukins) in cultures comparing to the unstimulated controls (Fig. 7). In the initial PD-1^+^/TIM-3^+^ T cell cultures, PD-1^+^ and PD-1^+^TIM-3^+^ relative cell counts, but not TIM-3^+^PD-1^−^ T cells, were incremented under the stimulation with anti-CD3+IL-2 comparing to the unstimulated controls. Besides, the relative increment in anti-CD3-stimulated initially TIM-3^+^ or PD-1^+^ T cells was significantly lower compared to their counterparts in the initially PD-1/TIM-3-negative cultures (with the exception of CD8^+^PD-1^+^TIM-3^−^ subset). Simultaneously, the combination of cytokines failed to induce a substantial increase in the initially PD-1- or TIM-3-positive T cell subsets (Fig. 8).

Thus, the stimulation with common γ-chain cytokines up-regulates PD-1 and TIM-3 during the proliferation of initially PD-1/TIM-3-non-expressing T lymphocytes, but does not lead to the expansion of PD-1- and TIM-3-expressing cells of MM patients *in vitro*.

## Discussion

Clinical trials of PD-1 inhibitors in RRMM were interrupted due to frequent adverse events and reduced survival rates [5,6]. The unsatisfactory response to the targeted immunotherapy may be due to excessive activation of T cells following PD-1 blockade, as the PD-1-expressing pool includes both exhausted and activated functional T cells [15,16]. Besides, T cells transiently up-regulate or at least maintain the expression of inhibitory checkpoint receptors (PD-1, TIM-3, TIGIT, LAG-3) following autologous and allogeneic HSCT [10–14], apparently as a result of common γ-chain receptor cytokine-mediated homeostatic proliferation [8–10]. In our present work, we comparatively studied the expression of common γ-chain receptors and associated intracellular molecules in PD-1- and TIM-3-negative and positive T cells at early post-transplant in MM patients.

As in previous studies [10–13], frequencies of PD-1- and TIM-3-expressing CD4^+^ and CD8^+^ T lymphocytes were significantly higher at the time of leukocyte recovery compared to the levels before AHSCT, due to a potent increment in T cell proliferative activity. Of note, the rate of proliferating cells was higher in the PD-1^+^TIM-3^−^ subset (but not in the TIM-3-expressing compartment) compared to PD-1/TIM-3-negative T lymphocytes.

Then, we assessed the expression of CD25, CD122, CD127 cytokine receptors and pSTAT5, which may reflect a functional capacity and cytokine responsiveness of PD-1^+^ and TIM^+^ T lymphocytes under conditions of homeostatic proliferation.

In general, frequencies of certain CD25/CD122/CD127- and pSTAT5-positive cells and directions of their changes in PD-1- and TIM-3-expressing T cell subsets following AHSCT coincided with PD-1/TIM-3-negative T cell compartment. Both CD4^+^ and CD8^+^ T lymphocytes expressed homeostatic cytokine receptors and pSTAT5 irrespectively of PD-1 and TIM-3 expression before the conditioning and at the engraftment and therefore seemed to be able to respond to homeostatic cytokine stimulation.

At post-transplant, the main changes were the decrease in CD127^+^ cells in virtually all studied subsets and the increment in CD122^+^ cells in PD-1^−^TIM-3^−^ and PD-1^+^TIM-3^−^ CD8^+^ T cells. “Physiological” CD127 down-regulation is well-described upon IL-7 stimulation during homeostatic proliferation [17,18]. Expression of CD122 is also up-regulated upon IL-mediated T cell activation [19–21].

Simultaneously, there were several distinctions between PD-1^+^ and TIM-3^+^ T lymphocytes in cytokine receptor expression. Before the conditioning, PD-1^+^TIM-3^−^ CD4^+^ and CD8^+^ T cells contained lower frequencies of CD127^+^ cells compared to both PD-1^−^TIM-3^−^ “conventional” T lymphocytes and TIM-3^+^ compartment. In previous studies, CD127^low/−^PD-1^hi/+^ cells had been determined as either an exhausted or an activated subset [22–25], since TCR-activated T cells also are considered to downregulate IL-7 receptor ɑ-chain (CD127) and up-regulate PD-1 expression [26,27].

Frequencies of CD25^+^ and CD122^+^ cells were higher within TIM-3-positive T cells compared with PD-1/TIM-3-non-expressing and PD-1^+^TIM-3^−^ subsets both before the conditioning and at the engraftment (non-significant for CD8^+^ T cells). CD25 expression is controlled by pSTAT5 and is observed when T lymphocytes are activated through the TCR and during homeostatic proliferation. Expression of CD122 appears to be induced with transcription factors T-bet and EOMES [28–30], both involved in T cell homeostasis, activation and exhaustion [30–32]. We speculate, that higher counts of cytokine receptor expressing cells in TIM-3^+^ T cell compartment denote the prevailing implication of TIM-3 in the regulation of homeostatic proliferation of T lymphopcytes, while the up-regulation of PD-1 is linked to the control of activation through TCR.

In our study, transcription factor EOMES was implicated predominantly in the CD8^+^ T cell compartment, which is consistent with literature data [28, 30–32]. Eomesodermin expressing CD8^+^ T lymphocytes were present in both PD-1/TIM-3-negative (at median 39%) and positive (at medians 40%–65%) subsets. At post-transplant, EOMES-expressing CD8^+^ T cell subsets increased even more, irrespectively of PD-1 and TIM-3 surface expression. The up-regulation of EOMES in CD8^+^ T cells under lymphopenic conditions seems to be “physiological” [30], and emphasizes a functional state of PD-1-/TIM-3-expressing CD8^+^ lymphocytes at post-transplant.

In CD4^+^ T cells, relatively moderate proportions (at medians ~30%) of PD-1/TIM-3^+^ subsets express EOMES. According to a few publications about EOMES expression in CD4^+^ T lymphocytes, its up-regulation has been associated with a highly inflammatory phenotype in murine models and patients with autoimmune diseases [33,34]. Surface expression of inhibitory molecules PD-1 and TIM-3 could be essential for the control of inflammatory and autoagressive CD4^+^ T cells, especially under the conditions of peripheral lymphocyte expansion. Besides, PD-1^+^EOMES^+^ CD4^+^ T cells are considered to be IL-10-producing T regulatory type 1 cells [34,35], a still poorly studied population. Its quantitative and functional characteristics as soon as the biological and clinical significance of checkpoint receptor expression need to be determined.

We have shown that CD25^+^ CD4^+^ T lymphocytes did not express EOMES. For other homeostatic cytokine receptors, no such relationships were found. Most likely, the co-expression of CD25 and checkpoint receptors may be an indicator of proliferating functional (not exhausted) CD4^+^ T cells, and activated natural regulatory T cells (nTreg). It is well-established that nTregs constitutively express CD25. Therefore, this T cell population appears to be EOMES-negative. “Exhaustion” of nTregs and expression of checkpoint molecules are mediated by distinct pathways, and their functions require thorough investigations.

Various research groups have shown an increment in PD-1^+^ and TIM-3^+^ T lymphocytes upon stimulation with common γ-chain cytokines [8–10]. Nonetheless, it was uncertain, whether homeostatic cytokines induced PD-1 and TIM-3 expression on initially PD-1/TIM-3-negative T lymphocytes or caused the expansion of pre-existing PD-1/TIM-3-positive subsets. To assess it, we stimulated magnetically isolated PD-1^−^TIM-3^−^ and PD-1^+^ and TIM-3^+^ CD3^+^ T cells of MM patients with the combination of cytokines IL-2, IL-7, IL-15 or with anti-CD3 and IL-2 *in vitro*. The stimulation with anti-CD3 increased the proportions of all PD-1^+^ and TIM-3^+^ subsets in initially PD-1 and TIM-3 non-expressing T cell cultures. Relative counts of PD-1^+^ and PD-1^+^TIM-3^+^ CD4^+^ and CD8^+^ T cells were also incremented in the initially PD-1/TIM-3-expressing cultures following anti-CD3 stimulation while there was no increase in TIM-3^+^ subsets. Up-regulation of checkpoint receptors following TCR-mediated activation is well established [15]. The diminished anti-CD3 stimulated proliferation of initially TIM-3-expressing T lymphocytes might be linked with more profound exhaustion within this subset compared to the PD-1-expressing compartment [7].

The stimulation with homeostatic cytokines also up-regulated PD-1 and TIM-3 during the proliferation of initially PD-1/TIM-3-non-expressing T cells but failed to expand PD-1^+^ and TIM-3^+^ subsets *in vitro*. To our knowledge, this is the first time to show a selective effect of common γ-chain cytokines on checkpoint receptor expression within PD-1/TIM-3-negative T lymphocytes. The unresponsiveness of PD-1 and TIM-3 expressing T cells to cytokine stimulation may either reflect their dysfunctional/exhausted state or—quite the opposite—be a manifestation of so-called “homeostatic inhibition” [10], which impedes the excessive expansion of fully functional activated lymphocytes. According to the obtained *in vitro* data, we can speculate that combined administration of IL-2 drugs with prolonged half-life and PD-1 inhibitors (as soon as future anti-TIM-3 MAbs) may facilitate effector T cell reinvigoration upon checkpoint blockade. Recent murine studies have also established that IL-2 can dramatically ameliorate the efficacy of anti-PD-1 treatment. The effect has been achieved due to overcoming the unresponsiveness of PD-1^+^T Cell Factor^+^ stem-like CD8^+^ T cells with the subsequent generation of functional CD8^+^ T cells. The authors emphasized the involvement of CD25 receptor binding in anti-PD-1 mediated T cell expansion [36,37]. In the same way, checkpoint blockade appears to alleviate lymphopenia-induced proliferation of PD-1^+^ T cells at early post-transplant. Nevertheless, such T cell expansion may raise the possibility of immune-mediated adverse events.

### Limitations

Here, we did not evaluate the up-regulation of the inhibitory receptors in various populations of regulatory T cells - nTregs, type 1 regulatory T cells and poorly studied CD8^+^ Tregs, - which is a limitation of our study. It should be emphasised, that nTregs are known to constitutively express CD25 and down-regulate CD127, as soon as CD8^+^ Tregs are CD122-positive. The analysis of PD-1/TIM-3-expressing Tregs was not the scope of the study and requires further research. Another limitation is the absence of data on bone marrow PD-1/TIM-3-expressing T cells at the engraftment, but this source is not available closely after AHSCT.

## Conclusion

Our data showed that substantial counts of PD-1^+^ and TIM-3^+^ T lymphocytes expressed common γ-chain cytokine receptors and pSTAT5; fluctuations in their frequencies at early post-transplant coincided with PD-1/TIM-3^−^ T cell compartment. Checkpoint receptor expressing T cells appear to be able to respond to cytokine stimulation. Simultaneously, a higher proportion of TIM-3^+^ T cells express homeostatic cytokine receptors compared to PD-1^+^TIM-3^−^ subsets, which may reflect the differences in the functional state of PD-1- and TIM-3-expressing cell populations. Considerable proportions of CD8^+^ T cells (both PD-1- and TIM-3-negative and positive) express EOMES. Only moderate frequencies of CD4^+^ PD-1^+^/TIM-3^+^ T lymphocytes up-regulate this transcription factor. Besides, the surface presence of CD25 and intranuclear expression of EOMES in CD4^+^ T cells were mutually exclusive regardless of PD-1 and TIM-3 expression, therefore CD4^+^CD25^hi^CD127^−^FOXP3^+^ nTregs appear to be EOMES-negative. The stimulation with common γ-chain cytokines up-regulates PD-1 and TIM-3 during the proliferation of initially PD-1/TIM-3-negative T cells, but does not lead to the expansion of PD-1^+^ and TIM-3^+^ subsets of MM patients *in vitro*. The combined administration of extended forms of IL-2 and checkpoint inhibitors can facilitate effector T cell reinvigoration. Checkpoint blockade seems to relieve lymphopenia-induced proliferation of PD-1^+^ T lymphocytes at early post-transplant, while such T cell expansion may raise the possibility of immune-mediated adverse events.

## Data Availability

Some data analyzed during the current study contain personal medical information and are available from the corresponding author on reasonable request.
